# Effects of eco-friendly carbohydrate-based superabsorbent polymers on seed germination and seedling growth of maize

**DOI:** 10.1098/rsos.171184

**Published:** 2018-02-14

**Authors:** Jinghe Tao, Wenxu Zhang, Li Liang, Ziqiang Lei

**Affiliations:** Key Laboratory of Eco-Environment-Related Polymer Materials of Ministry of Education, Key Laboratory of Polymer Materials of Gansu Province, College of Chemistry and Chemical Engineering, Northwest Normal University, Lanzhou 730070, People's Republic of China

**Keywords:** superabsorbent polymers, carbohydrate, seed germination, seedling growth, maize

## Abstract

Desertification is the degradation of land in arid and semi-arid areas. Nowadays, lack of water and desertification are extreme problems for plant survival and growth in the arid and semi-arid areas of the world. It becomes increasingly important as to how to let the plant absorb moisture more effectively for keeping growth strong. We synthesized superabsorbent polymers (SAPs) with carbohydrate and characterized them by Fourier transform infrared spectra analyses, scanning electron microscopy and thermogravimetric/differential thermal analyses. Then, a completely randomized experiment was conducted to assess the effect of carbohydrate-based SAPs on seed germination and seedling growth of maize in an artificial climate chest. The results showed that adding an appropriate amount of SAPs could improve root length, shoot length, total biomass, germination potential and germination rate. It indicates that this SAP is not toxic to plants and can promote seed germination, and at the same time provides a possibility of replacing other substrates.

## 1. Introduction

Maize (*Zea mays* L.) is widely planted in tropical and temperate regions around the world. Also, it is the second largest crop in China, which is planted almost all over the country every year. Nonetheless, maize needs a lot of water in order to grow.

China, especially the northwest arid and semi-arid areas of China, is one of the countries facing serious shortage of water resources. Spring drought is the key limiting weather factor to the maize seedling situation [1]. Research has shown that drought hinders the process of seed germination [2], reduced the seed germination rate (GR) and inhibited the roots of nutrient absorption and seedling growth development [2,3].

Therefore, to improve agricultural water use, ease the difference between the supply and demand of water resources and develop high-efficiency water saving becomes the principal measure for sustainable development of agriculture [4]. It is possible that the application of a water retention agent and chemical drought resistance water saving technology can effectively solve the problem [5]. Superabsorbent polymer (SAP) is known for its ultra-strong water absorbing power. It can quickly absorb tens or even thousands of times the weight of liquid water [6], mainly owing to its three-dimensional network and large number of hydrophilic groups [7]. According to the existing research reports, SAP has been applied in various fields, such as health supplies [8,9], medical materials [10,11], sewage treatment [12] and agriculture [13]. In recent years, SAP as a soil modifier has shown beneficial effects in desertification soil restoration and soil water conservation in arid areas [14]. It can effectively improve soil moisture content, increase soil porosity, improve the soil structure, increase soil fertilizer effect [14,15] and improve the survival rate of plants and increase production [16]. Nonetheless, it has not been reported that SAPs have been applied on plants directly. Different preparation methods, raw materials, experimental conditions and other factors cause tremendous gaps in the performance of SAPs [17,18]. Therefore, it is very important for us to prepare a SAP which is easy to degrade, eco-friendly, causes strong water retention, has abundant raw materials and involves low cost. Also, it is necessary to study its application of methods and effects on agriculture.

Hence, the main objectives of this study were: (i) to assess the water retention performance of the SAPs, which were prepared with carbohydrate by our laboratory; (ii) to determine the effects of SAPs in improving seed germination and seedling growth of maize; (iii) to ascertain the most suitable application amount and application method for planting maize that could make maize grow better; and (iv) to extend the usefulness of carbohydrate polymers.

## 2. Material and methods

### 2.1. Materials

Maize straw (MS), acrylic acid (AA), *N*,*N*-methylenebisacrylamide (MBA), potassium persulfate (KPS), palygorskite clay (PGS) and maize seeds (MS, Beijing Baofeng Seed Co., Ltd, Beijing, China) were used. Before carrying out the experiment, the healthy and uniform seeds were sterilized with 4% sodium chloride solution for 30 min and diluted in the same solution 10 times, followed by repeated washing with distilled water. All reagents are of analytical grade and double distilled water is prepared in the laboratory itself.

### 2.2. Preparation of superabsorbent polymers

The preparation method of SAPs is as follows: first, MS was dispersed by mechanical agitation in a 250 ml three-necked flask filled with a proper amount of distilled water. The reactor was in a water bath at 70°C, and was stirred for 1 h to complete the gelatinization reaction. The chain of MS was extended as far as possible to ensure a follow-up reaction is carried out. Then, neutralized AA, 0.4 g PGS and 0.03 g cross-linking agent MBA were mixed and added into a three-necked flask for further reaction. Finally, 0.1 g KPS under nitrogen protection polymerization was added and kept for 30 min for the reaction to complete. The samples were dried in a drying oven at 70°C until a constant weight was achieved. Then samples were milled and passed through 70 mesh sieves to make them the same size for further use.

### 2.3. Method of characterization

#### 2.3.1. Fourier transform infrared spectra analysis

The samples were analysed by Fourier transform infrared (FTIR) spectrometer (FTIR-FTS3000) at the wavenumber of 400–4000 cm^−1^ and the samples dispersed in potassium bromide pellets.

#### 2.3.2. Thermogravimetric analysis

The thermogravimetric analysis was examined on an America TA Company Instruments (TGAQ100) at a temperature range of 0–600°C and with a heating rate of 10°C min^−1^ under an atmosphere of nitrogen.

#### 2.3.3. Morphology analysis

The surface morphology and pore morphology of the sample was researched using the field emission scanning electron microscopy (FE-SEM, Ultra Plus, Carl Zeiss) at an accelerating voltage of 5.0 KV after coating the sample with a gold film.

### 2.4. Swelling and water retention investigation

The measurement of the equilibrium water absorption was carried out as follows: under room temperature, some dry samples were immersed in excess distilled water to reach an equilibrium of swelling. The excess water in the swollen SAP was then filtered through a gauze bag. Then, the equilibrium in water absorption is calculated using the following formula:

$$Q_{\mathrm{eq}}=\frac{\left(w_{2}-w_{1}\right)}{w_{1}},$$

where *W*_1_ is the dry weight of SAPs. *W*_2_ is the swollen weight of SAPs, and *Q*_eq_ is the equilibrium water absorbency of SAPs.

The water retention property is determined by the following steps: the swollen SAPs are placed into the oven at 35°C. At regular intervals they are weighed and recorded. The following formula is used to calculate the water retention at different times:

$$Wr=\frac{w_{3}}{w_{2}}\times 100\%,$$

where *W*_3_ is the weight of swollen SAPs at different times on different temperatures.

### 2.5. Germination test

The completely randomized experiment was designed with three replicates. Two treatment methods were considered: the swollen SAPs and no-treatment SAPs. The treatment of SAPs was set at three levels, with five experimental groups, namely A, B, C, D and E (table 1). There was a certain amount of distilled water, different treatments of the SAPs and two pieces of filter paper wetted with distilled water in each culture dish as different conditions of cultivation of maize. Pre-treated maize seeds were washed two times with distilled water, and then sprinkled on prepared culture dishes. There were 30 maize seeds in each culture dish. It was tested in an artificial climate chamber (RQH-280C, Shanghai Kuangbei Industrial Co., Ltd) which created a growth environment (table 2).

**Table 1:** The experimental design and treatment.

treatment	water (g)	swollen SAPs (g)	no-treatment SAPs (g)
A	21	0	0
B	0	21	0
C	0	32	0
D	21	0	0.055
E	32	0	0.082

**Table 2:** Set parameters of the artificial climate chamber in the experiment.

setting parameters	part one	part two
temperature	20°C	30°C
humidity	40%	25%
light intensity	0	4
time	720 min	720 min

### 2.6. Germination rate and germination potential

GR was calculated using the following formula:

$$\text{GR}(\%)=\frac{G_{t}}{T}\times 100,$$

where *G_t_* is the number of germinated seeds at time *t* (it is usually different germination days and it represents the last germination day in this literature), and *T* is the total number of tested seed.

Seed germination potential was an important indicator of testing seed quality. It could be used to represent seed vitality which incorporates the uniformity of germination and the formation of seedlings. It was expressed in the following formula as follows:

$$\mathrm{GP}(\%)=\frac{G_{t}}{T}\times 100,$$

where GP is germination potential, *G_t_* is the number of germinated seeds at time *t* (it is not an accumulated number, while it represents the number of germinated seeds observed at the corresponding time), and *T* is the total number of tested seed.

### 2.7. Water consumption

The weight of each culture dish was measured at regular intervals, and then according to the water consumption in each dish, enough distilled water was added to maintain a constant weight. Then, according to the water consumption and the amount of water added the ability of SAPs to retain water in each experiment was assessed.

### 2.8. Root length, shoot length and biomass

The length of the root and the shoot of maize seedlings in each experiment were measured at 7 days post-treatment separately. The dry weight of root and shoot was measured after maintaining a constant temperature in a drying oven at 105°C for 8 h.

### 2.9. Statistical analyses

Statistical analyses were conducted using the computer software SPSS v. 19.0 for Windows (SPSS, Inc., Chicago, IL, USA). Analysis of variance (ANOVA) was used to compare priming treatment effect, and significant differences of means were separated using Duncan's test (*p *< 0.05).

## 3. Results and discussion

### 3.1. FTIR spectra analysis

**Figure 1.**
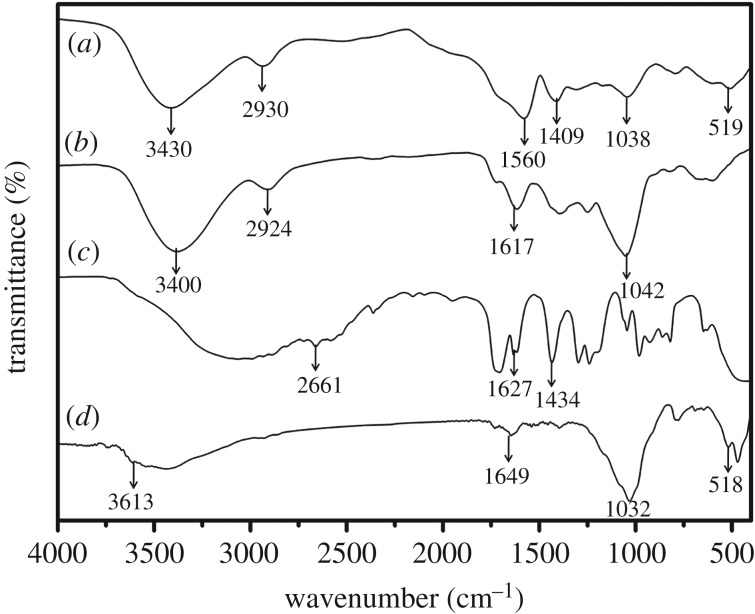
FTIR spectra of (*a*) SAPs, (*b*) MS, (*c*) AA and (*d*) PGS.

From the FTIR spectra of MS, it can be seen that at 2924 cm^−1^ there are characteristic peaks owing to the C–H stretching vibration [19]. Compared with the infrared spectrum of MS (presented in figure 1*d*), at 2930 cm^−1^ samples showed a change in migration and a change in the intensity of the C–H peaks. Peaks at 3613 and 1649 cm^−1^ are formed by stretching and bending vibrations of OH groups, respectively, on the surface of PGS, which can be seen in the diagram and disappear after reaction. At the same time, there are some new peaks at 1560 and 1409 cm^−1^, which are attributed to the asymmetric stretching and symmetric stretching of –COO^−^ groups [20]. The other characteristic peaks in the range of 470–779 cm^−1^ are assigned to the bending vibration of M–O (M represents Si or other metal cations existing in PGS) [21], these peaks also weaken after reaction. In combination with FTIR spectra analysis, polyacrylic acid chains have been successfully grafted into the MS macromolecular chains. PGS also participates in graft polymerization via surface hydroxyl groups.

### 3.2. Thermogravimetric analysis

**Figure 2.**
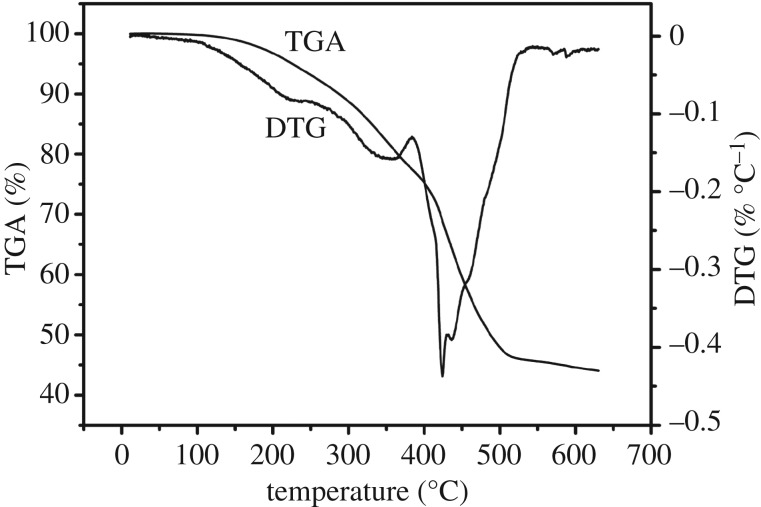
Thermogravimetric/differential analysis (TGA/DTG) of SAPs.

Figure 2 represents thermogravimetric analysis curves of SAPs we synthesized. It can be observed in the figure that it is a multi-step degradation process. First of all, from the thermogravimetric analysis curves, it is found that the sample has a slight mass loss between 0 and 243°C, which is probably caused by the loss of water or volatility of other compounds [22]. Then, at a temperature range of 243–384°C, a 16 wt% weight reduction may be owing to the removal of CO_2_ molecules from the polymer backbone. In addition, the maximum degradation temperature is 424°C. This can be attributed to the removal of water molecules from the two adjacent carboxylic groups of the polymer chains, resulting in the formation of anhydride [23], followed by the main chain fracture and the breakdown of the cross-linked network structure [17,24]. Finally, there are traces of mass loss at temperatures exceeding 550°C, mainly because the decomposed part will be further decomposed.

### 3.3. Morphology analysis

**Figure 3.**
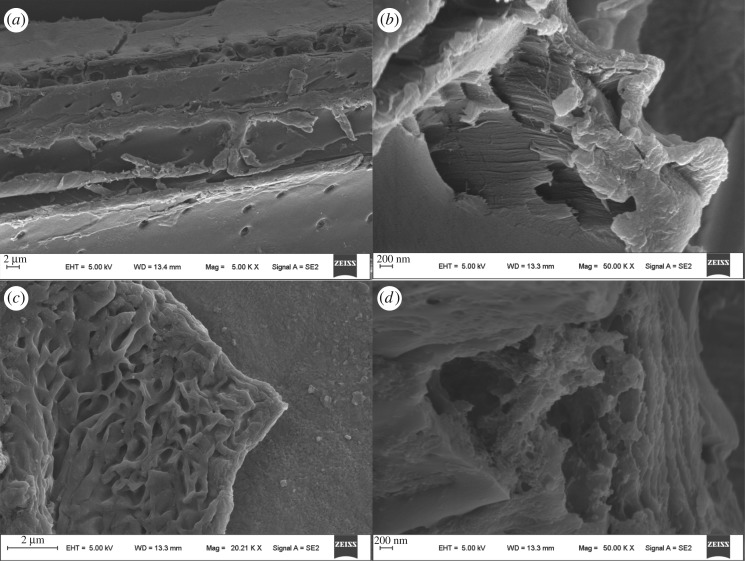
Scanning electron microscopy of MS (*a,b*), and SAPs (*c,d*).

The scanning electron microscopy of SAPs in our compound and MS are presented in figure 3. Obviously, the surface morphology of carbohydrate-based SAPs (figure 3*c,d*) is different from that of MS (figure 3*a,b*). The images show that MS has a dense and smooth surface and generally presents rods. It is obvious that the SAPs present a loose and porous wrinkled surface, and many creases and holes can be observed; this kind of interconnected pore can make SAPs absorb and retain water more easily [25]. This surface is useful for penetration of water into the polymeric network, and benefits water absorbency of corresponding SAPs [26]. The pores can retain moisture at the same time and provide plant growth. Also, it can provide sufficient air for the plant roots.

### 3.4. Swelling and water retention investigation

**Figure 4.**
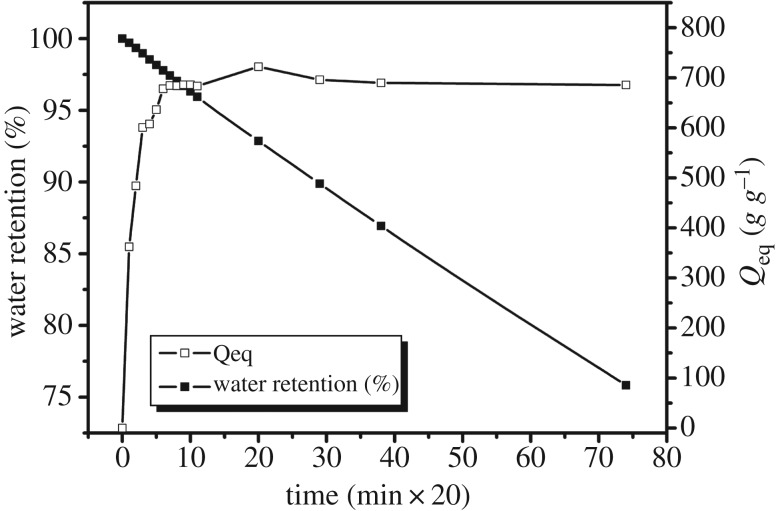
Water absorbency (white squares) and water retention (black squares) of SAPs.

The water absorption and water retention of the SAPs can be clearly seen in figure 4. As shown in the figure, more water is absorbed by the SAPs as time increases. After 3 h, its water absorbency hardly changes, indicating that it has reached a state of balance. This process of rapid water absorption is beneficial to the collection of water. As time goes by, water evaporates and water retention gradually decreases. It can be seen from the figure that the water retention curve is a relatively flat curve, indicating that the evaporation rate is slow. This is primarily owing to the fact that the hydrophilic groups adsorb water molecules which are immobilized in the three-dimensional cross-linked network [7]. The water can be retained for 4 days at 35°C, which means that the SAPs have a good water holding capacity. Excellent water retention can store the water better, thus providing plants with sufficient moisture. It can serve as a small reservoir to provide water for plants.

### 3.5. Germination rate under different experimental treatments

**Figure 5.**
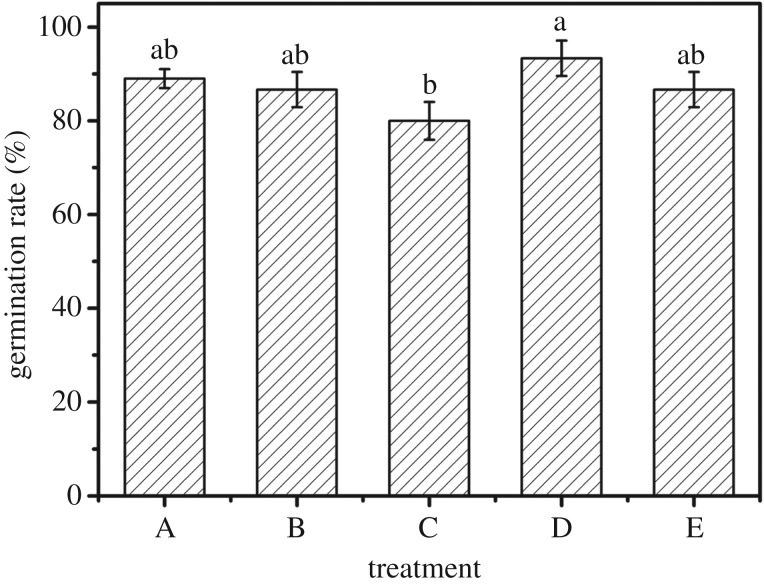
GR under different experimental treatments: (A) water, (B) one-third swollen SAPs, (C) moiety swollen SAPs, (D) one-third no-treatment SAPs, water, and (E) moiety no-treatment SAPs, water. Each value represents the mean ± s.e. (*n* = 30). The different lowercase letters indicate significant differences (*p* < 0.05) among treatments as determined by Duncan's test.

It is clearly seen that the GR of maize varied with SAPs application amount and method (figure 5). Compared with treatment A (89%), the GR of maize in treatment B, treatment C and treatment E which is 86.67%, 80% and 86.67% respectively is slightly lower (*p *> 0.05). In all of these experiments, the GR of treatment D is the highest (93.33%). With the experimental results, it can be seen that both added swollen SAPs and no-treatment SAPs, GR of maize change is not manifest. According to the statistics, the GR of the addition of SAPs did not show a significantly inhibitory effect. Also, the addition of no-treatment SAPs has improved the GR. Previous studies reached a similar conclusion [16]. From what has been discussed above, it can be concluded that effects of SAPs on germination of maize seed and maize seedling growth do not produce toxins [27]. It even improves the GR slightly. It also proves that SAPs can be applied to the seed germination and seedling growth, and it provides a feasible use for the application of SAPs in agriculture.

### 3.6. Germination potential under different experimental treatment

**Figure 6.**
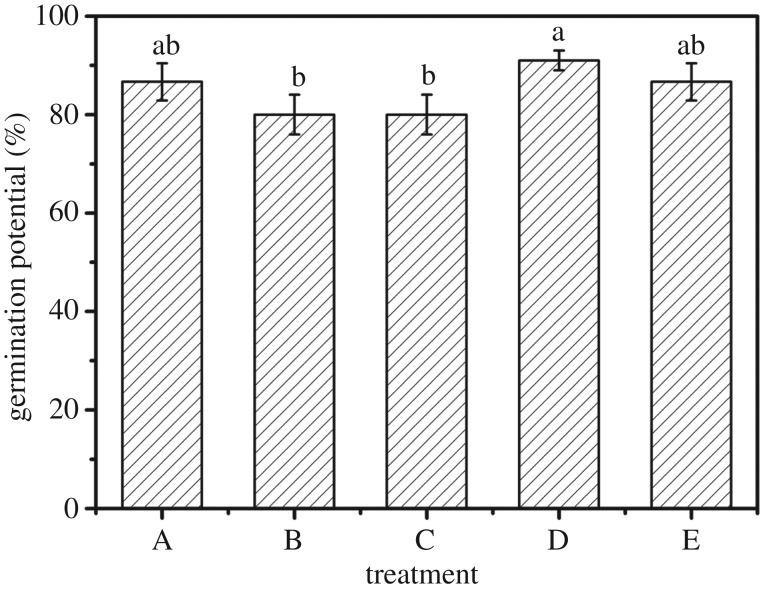
Germination potential under different experimental treatments: (A) water, (B) one-third swollen SAPs, (C) moiety swollen SAPs, (D) one-third no-treatment SAPs, water, and (E) moiety no-treatment SAPs, water. Each value represents the mean ± s.e. (*n* = 30). The different lowercase letters indicate significant differences (*p* < 0.05) among treatments as determined by Duncan's test.

It is demonstrated that the germination potential of maize under different experimental treatments had a few distinguishing factors (figure 6). The germination potential of maize from treatment B and treatment C are both 80%, slightly below (*p *> 0.05) that of treatment A. The germination potential of maize in treatment D is evidently more than those of treatment B and treatment C (*p *< 0.05).

According to the research results, by adding swollen SAPs the germination potential of maize is slightly lower. It indicates that swollen SAPs slightly inhibit the germination of maize, while adding no-treatment SAPs maize germination potential showed a small increase. The no-treatment SAPs have some slight advantages advantages for corn germination potential. The possible causes of these phenomena are that although the swollen SAPs contain water, the water absorbed by SAPs is not able to be immediately released for maize seed use when it comes to germination. The first step in seed germination is that the seed absorbs enough water to keep it in a saturated state, then it will germinate in appropriate conditions. In consequence, adding swollen SAPs has a capacity to provide water, but it is difficult to reach the saturated state which is needed for seed germination. As a result of this, germination potential has some influence. While adding no-treatment SAPs, because the moisture is not absorbed by SAPs the water can be directly used by the seed and SAPs can reserve more water for plant germination by absorbing excess moisture and reducing the amount of direct evaporation [28].

### 3.7. Water consumption under different experimental treatments

**Figure 7.**
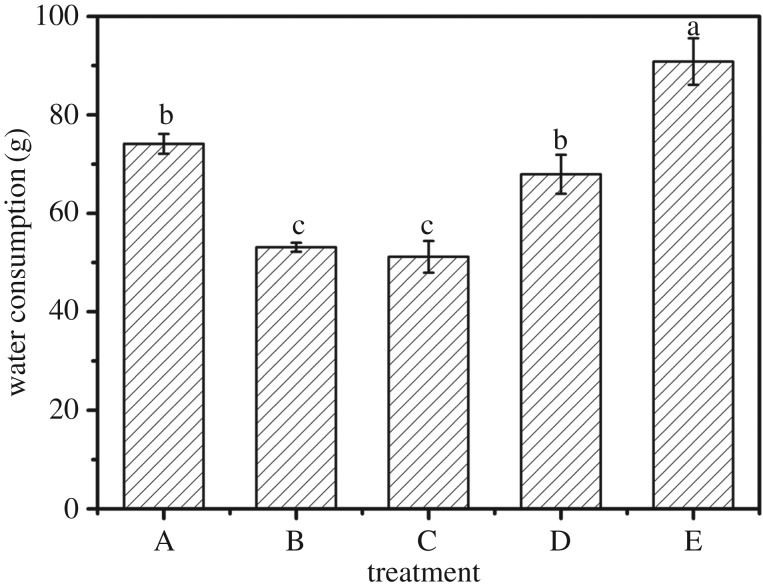
Water consumption under different experimental treatments: (A) water, (B) one-third swollen SAPs, (C) moiety swollen SAPs, (D) one-third no-treatment SAPs, water, and (E) moiety no-treatment SAPs, water. Each value represents the mean ± s.e. (*n* = 30). The different lowercase letters indicate significant differences (*p* < 0.05) among treatments as determined by Duncan's test.

It was clearly shown that the water consumption of maize has apparent differences under the different treatments (figure 7). The water consumption of maize in treatment B and treatment C which are 53.10 and 51.15 g is lower (*p *< 0.05) compared to that of treatment A (74.12 g). Moreover, water consumption in treatment E (90.82 g) is significantly higher than those of other experiments (*p *< 0.05).

From the results, we can see that the use of swollen SAPs has a significant role in water retention. This confirms the conclusion that SAPs have a strong power of water absorption and water retention. The water it absorbs and retains could be used to release water slowly during germination and growth of maize. According to the results of previous studies, the water absorptive capacity of plant roots is greater than that of water retention, therefore the water absorbed by the SAPs can supply the plant [29]. Compared with the control group, adding the swollen SAPs consumes less water. This is mainly because swollen SAP itself contains a certain amount of water. Because this part of the water is absorbed, it is not directly evaporated during the growth of maize. That is to say direct reduction of water evaporation plays a role in retaining moisture. When the maize is growing, it will use the nearest water which SAPs contain. When this water is gradually absorbed as time goes by, the remaining water in swollen SAPs is released slowly, like releasing fertilizer [30,31] and supplies water for plant growth. Therefore, in similar conditions, adding the swollen SAPs could reduce the water consumption and have an outstanding water saving effect. According to previous studies, it is known that 95% of their stored water is available for plant absorption [32].

### 3.8. Root length and shoot length under different experimental treatments

**Figure 8.**
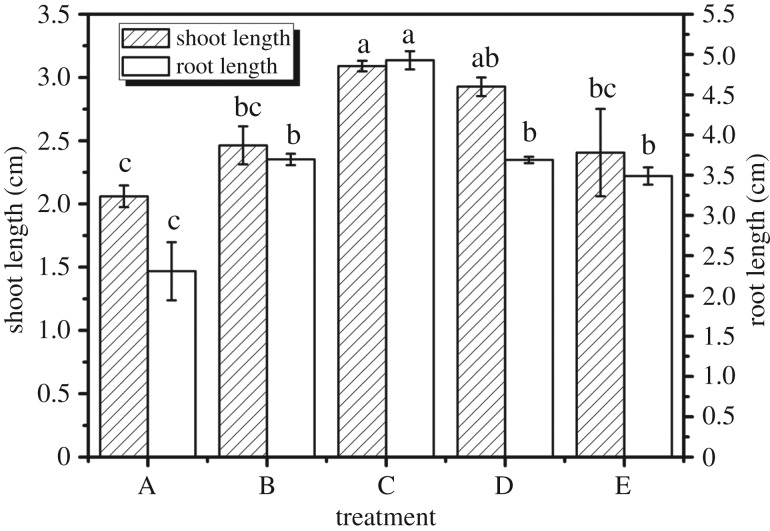
Root length and shoot length under different experimental treatments: (A) water, (B) one-third swollen SAPs, (C) moiety swollen SAPs, (D) one-third no-treatment SAPs, water, and (E) moiety no-treatment SAPs, water. Each value represents the mean ± s.e. (*n* = 30). The different lowercase letters indicate significant differences (*p* < 0.05) among treatments as determined by Duncan's test.

As depicted in figure 8, the shoot length of the maize (3.09 cm) significantly increased with treatment C over the control (*p *< 0.05). Concretely, there are obvious changes in different experimental treatments. In treatment A, the shoot length (2.06 cm) significantly decreases compared to other experiments after 7 days (*p *< 0.05). Treatment D is 2.93 cm which is slightly higher than in treatment B and treatment E (*p *> 0.05), while it is slightly lower than treatment C (*p *> 0.05).

It can be seen from the results of the study that compared with the control, (treatment A) the shoot length of adding SAPs to the experimental group increased. It shows that SAPs promote the growth of maize seedlings. Experiment C's shoot length is the longest in all of the experiments. It is quite clear from this that SAPs play a promoting role in the growth of shoot length. This also illustrates that SAPs have a promotion function in the root.

The root length of maize varied with application amount and application method. For treatment C, the root length is significantly higher (4.93 cm) than that of the control after 7 days (*p *< 0.05), indicating a significant stimulation. For treatment A, the root length of maize is 2.31 cm, significantly lower than other experiments (*p *< 0.05).

It can be seen from figure 8 that root which has added SAPs is significantly longer than root which has no added SAPs. In all experiments, experiment C has the longest root length. It is hence not difficult to see that SAP has an obvious promoting role in the process of the growth of maize root and the promoting effect of an appropriate amount of swollen SAPs is more obvious. This may be owing to swollen SAPs containing different types of ions and on account of its hydrophilic and carboxylic groups [33]. This ion interaction could stimulate the growth of roots, after all, a lot of research has shown that low concentrations of elements can stimulate the growth of plants [34–36].

### 3.9. The dry weight of aboveground biomass, belowground biomass and total biomass under different experimental treatments

**Figure 9.**
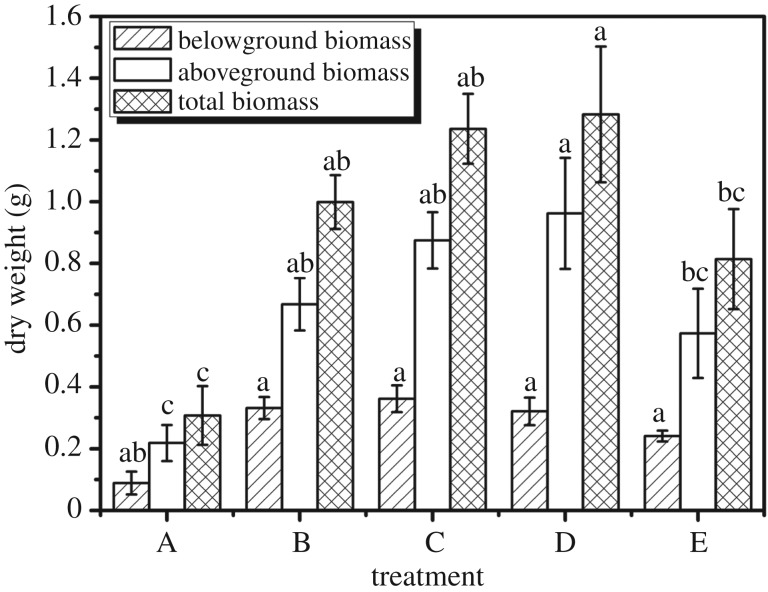
Belowground biomass, aboveground biomass and total biomass under different experimental treatments: (A) water, (B) one-third swollen SAPs, (C) moiety swollen SAPs, (D) one-third no-treatment SAPs, water, and (E) moiety no-treatment SAPs, water. Each value represents the mean ± s.e. (*n* = 30). The different lowercase letters indicate significant differences (*p* < 0.05) among treatments as determined by Duncan's test.

It is indicated that the different treatments have a significant impact on the aboveground biomass of maize (figure 9). The dry weight of aboveground biomass of maize in treatment B, treatment C and treatment D is 0.08, 0.09 and 0.11 g respectively, which is more than that of treatment A (0.03 g) (*p *< 0.05). In all treatments, the dry weight of aboveground biomass of treatment D is highest.

Based on the determination of aboveground biomass accumulation, we discover experiments which add SAPs have a significant promoting effect. The promoting effect of SAPs is slightly altered by the different dosage and state. The promotion in experiment D is the most obvious, it shows that it has stronger shoots. This is mainly because of the developed lateral roots, which could provide nutrition for the growth of aboveground parts from multiple channels.

In the influence of different treatments on belowground biomass of dry weight, treatment A (0.02 g) is the lowest. In addition to treatment A, dry weight had no significant change (*p* < 0.05).

The result shows that SAPs have a promoting effect on belowground biomass of maize by weight of the belowground biomass. The value in experiment C is highest. This is mainly because the plants have developed a main root. The more developed the main root in a plant, the easier the transport of nutrients and a better, accumulation of root biomass.

We can see the accumulation of total dry biomass by adding SAPs is better than the control. According to these data, we know that the difference between them is not very significant (*p *> 0.05); with regards to dry total biomass, treatment A is 0.05 g also far less than treatment B, treatment C and treatment D whose dry total biomass is 0.13, 0.13 and 0.15, respectively (*p *< 0.05). Treatment E is 0.06 g marginally better than the control (*p *> 0.05), while it is inferior evidently to treatment B, treatment C and treatment D (*p *< 0.05).

It can be observed in figure 9 that adding SAPs has a promoting effect on the dry weight accumulation. This can also be observed in other research [1,37,38]. Experimental D's dry weight accumulation reaches the maximum value in all experiments. In other words, the right amount of untreated SAPs is more conducive to the accumulation of material in these conditions. This is because the SAPs absorption of water is a dynamic process. The plant can directly obtain sufficient moisture before it reaches the swollen state. Then the swollen SAPs can release moisture slowly. It ensures that maize gets a better biomass accumulation. Just keeping the growth of the plants in the fundamental period needs enough water [39]. A lack of moisture will restrain the growth of the plants [40].

## 4. Conclusion

In this paper, a kind of SAP is prepared, and we apply it to the process of the germination of maize, analysing the effects of SAPs on germination and growth of maize through experiments on the application amount and application methods of SAPs. It is hence not difficult to observe that the most favourable treatment for plant growth is treatment D. In treatment D, the accumulation of total biomass, germination potential and GR is best and furthermore, water consumption is less. It indicates that carbohydrate-based SAPs are good for germination and growth of maize.

## Data accessibility

All relevant data in this research are presented in this paper.
